# Grit increases strongly in early childhood and is related to parental background

**DOI:** 10.1038/s41598-022-07542-4

**Published:** 2022-03-03

**Authors:** Matthias Sutter, Anna Untertrifaller, Claudia Zoller

**Affiliations:** 1 Max Planck Institute for Research On Collective Goods, Bonn, Germany; 2 University of Cologne, Cologne, Germany; 3 University of Innsbruck, Innsbruck, Austria; 4 Management Center Innsbruck, Innsbruck, Austria

**Keywords:** Developmental biology, Psychology

## Abstract

Grit has been identified as a very important non-cognitive skill that is positively related to educational achievements and labor market success. Recently, it has also been found to be malleable through interventions in primary schools. Yet, little is still known about its development in early childhood and the influence of family background. We present an experiment with 429 children, aged 3–6 years. We measure the level of grit as children’s perseverance in a real effort task and their willingness to challenge themselves successfully with another, more difficult task. Based on a principal component analysis, we find that grit increases strongly with age. Parents’ assessment of their child’s grit is correlated with the actual behavior of their child. Education of parents plays a role for perseverance. Yet, children’s level of patience is unrelated to their level of grit.

## Introduction

Non-cognitive skills have been identified as crucial for lifetime outcomes, such as good health, higher education, and labor market success^1–5^. Among these non-cognitive skills, grit has received particular attention in recent years. While grit is often defined as a combination of an individual’s perseverance of effort and the passion for a particular long-term goal, the definitions one can find in the literature differ in nuances^6–10^: grit is often related to an ability to work hard, to be persistent in a task, to challenge oneself and not give up even in case of intermediate drawbacks. Despite those mild differences in definitions, the literature has produced a coherent pattern about the importance of grit. It has been proven to be highly indicative of educational achievement^6,11,12^ as well as higher earnings, tenure and promotions in companies or innovativeness and success of entrepreneurs^13–15^. Because of its importance for later outcomes in life, recent work has examined how grit can be trained in 8- to10-year-old primary school children^8^. Yet, little is known so far about the development of grit in early childhood (prior to entering primary school) and how it relates to family background.

In this paper, we present an experimental study with 429 children, aged 3 to 6 years, to examine whether and to which extent grit increases with age and which factors drive its potential development. Pre-school age has been identified as a critical period for the development of economic preferences^10,16,17^, and of non-cognitive skills more generally^4^, for which reason we hypothesize that grit as an important non-cognitive skill develops over the same time frame.

Rather than using a single measure for grit as is often done in the literature^7,8^, we use a compound measure that combines three different choices of children, thus reducing potential measurement error^5,18^. We consider (i) perseverance as an ability to work hard, (ii) the willingness to challenge oneself by choosing voluntarily a more difficult task when given the choice, and (iii) the likelihood to follow through with that task until completion. Each of these aspects of grit has been found to be important both for educational achievements and labor market outcomes^12,13,19^. We can examine both the compound measure and each of its components separately. As grit has often been linked to time preferences^7,20^, we also consider this link as a robustness check.

Complementing children’s data with a survey among their parents allows studying the role of family background for the early development of grit. The family environment and parents’ characteristics have a large influence on various cognitive and non-cognitive skills^21–25^, but the relation of family background to the formation of grit in early childhood is still underexplored. So, in sum our paper is the first to present evidence on the development of grit in children as young as 3 to 6 years old, is able to present both a composite score of grit that captures many facets that are associated with grit, but at the same time can present evidence for the single components separately, and adds parental background and survey data to enrich our understanding of whether and to which extent grit develops in early age.

## Methods

We conducted our study in eight kindergartens in the city of Innsbruck, Austria, in spring 2017. The project was approved by the IRB of the University of Innsbruck (certificate of good standing 10/2017) and by the municipal authorities of the city of Innsbruck and all methods were performed in accordance with the relevant guidelines and regulations. Parents provided informed consent for their own and study participation of their children.

A total of 429 children between the ages of 3 and 6 (51% females) participated voluntarily (see Table S1 in the supplementary information, SI, for the number of boys and girls in each age cohort). We visited each kindergarten on two (or three – if the number of children was very large) consecutive days. All children were introduced one-on-one by a trained experimenter to what we call here the puzzle task, the perseverance task, and a time preference task. To ensure comprehension all children had to answer questions for each task (see SI for the experimental instructions and more details). Overall, 91% of the children could answer all control questions correctly, indicating that we succeeded in explaining the task so that even our youngest participants could understand them well. The final column in Table S1 in SI notes the fraction of children with complete understanding contingent on age. While this fraction tends to be higher in older children, the lowest fraction across all age groups is still high with 87% for 4-year-olds.

The results presented in the following remain robust to excluding all children who did not answer all control questions correctly. In each task, children could earn tokens which they could exchange for small presents (like balloons, hair clips, key chains, etc.). Anonymity was preserved by assigning children a code that could not be linked to their name.

In the puzzle task, children were presented with two puzzles that showed the same picture, but varied in the number of pieces and thus had different piece sizes. The experimenter called the puzzle with fewer pieces the easier one and the other puzzle with the larger number of pieces the more difficult one. Taking into account that the difficulty of doing such a task differs with age, we presented children aged 3 and 4 with an easy puzzle with 6 pieces and a difficult one with 12 pieces. For 5- and 6-year-olds we had one puzzle with 12 pieces, and the other one with 24 pieces (see Figure S.1 in SI for an illustration of the puzzles and its different versions). Children could choose which of the two puzzles they would like to have as a present. We take this decision as children’s willingness to challenge themselves, respectively to set ambitious goals (yes or no).

When explaining the puzzle task to children, we not only told them that they could keep the chosen puzzle for themselves. We also said that if they completed the puzzle correctly by the end of our visit on day one they would receive 1 token in case of the easy puzzle and 2 tokens for the difficult puzzle. The tokens were presented next to the respective puzzle to make the payoff difference salient. Importantly, we also stressed that working on the puzzle was voluntary, and that children could keep the puzzle even if they did not work on it during our visit. This way we use the completion of the puzzle as a child’s ability to follow through with this task (yes or no).

The perseverance task was a real effort task where children were asked to collect only yellow beads from a bowl of small, multicolored beads (see Figure S.2 in SI). To avoid any kind of peer effects, children were seated in separate cubicles. As a control for ability—which might (and actually does) develop with age—children were instructed to practice the task for 30 seconds. Afterwards, they were told to continue working on the task for as long as they wanted to. They were asked to notify the experimenter (by raising their hand) once they decided to stop working on the task. Then for each 20 yellow beads they had collected, they were given 1 token to be exchanged for presents. When describing the task, experimenters showed them a bowl with 20 yellow beads for visual reference and explained that this quantity was worth 1 token. In addition to measuring the number of beads, the experimenter also timed how long a child worked on the perseverance task. In the following, we will use the number of collected beads as outcome measure for perseverance, but in the SI we show that taking the time spent on the task yields the same qualitative results. After children had completed the perseverance task, we also asked them about how much they enjoyed the task (by pointing to different emojis to express their level of enjoyment) in order to control for how this aspect of the task might affect performance.

In the perseverance task, we had the following treatment variation. 188 children were assigned to an exogenous condition where 91 children were asked to do the task on the first day of our visit, and 97 to do it on the second day (but the explanation was always done on the first day). The remaining 241 children could choose between doing the task on the first or the second day. This treatment variation was motivated by a desire to examine whether giving children autonomy when to do a tedious task would impact their level of perseverance in that task. There is hardly any evidence for adults on how their productivity reacts to autonomy in choosing when to do what^26^, and we do not know any study for children on this aspect. We randomized children into the different treatments on the level of the classroom. In case of an uneven number of classes, we usually implemented for the last class the endogenous condition (where children could choose on which day to work on the task). We did so to ensure that we still would have a number of children in the lesser chosen option (of doing the task either today or tomorrow) that is comparable to the numbers in the exogenous conditions. In fact, 83 out of 241 chose to delay the task to tomorrow (while 158 chose today), which is close to the 91, respectively 97, children in the exogenous condition. It turns out that the number of beads collected does not differ significantly across conditions, with the exception that children who postponed the task to the second day in the endogenous condition worked significantly less than all others.

The time preference task let children choose among three options. In option 1 they received 2 tokens on the same day, but none on the next day. In option 2 they got 1 token today and 2 tokens tomorrow, and option 3 paid no tokens today, but 4 tokens on the next day. This implies that each token saved for the second day of our visit was doubled in its value. Children collected their tokens for the same day in one bag, and tokens for the next day (if any were chosen) were put in a separate bag with the child’s name written on it. The latter bags were returned to children the following day to allow children to exchange the saved tokens for additional presents. The number of tokens saved for tomorrow is our measure of a child’s patience.

In addition to collecting data from children, parents received a questionnaire asking for information on demographic variables, like number of siblings, parents’ workings status, education and household income, and parents’ assessment of their child’s perseverance, willingness to take a challenge, and patience (see SI). To assure anonymity, the parental survey contained only a code that allowed matching it with their child’s decisions.

## Results

In the following, we first present descriptive results on each of the three components of grit—perseverance, willingness to challenge oneself, and following through on the puzzle task—and then combine the three components by means of a principal components analysis (PCA) to get an encompassing measure of grit that does not rely on a single measure.

**Figure 1:**
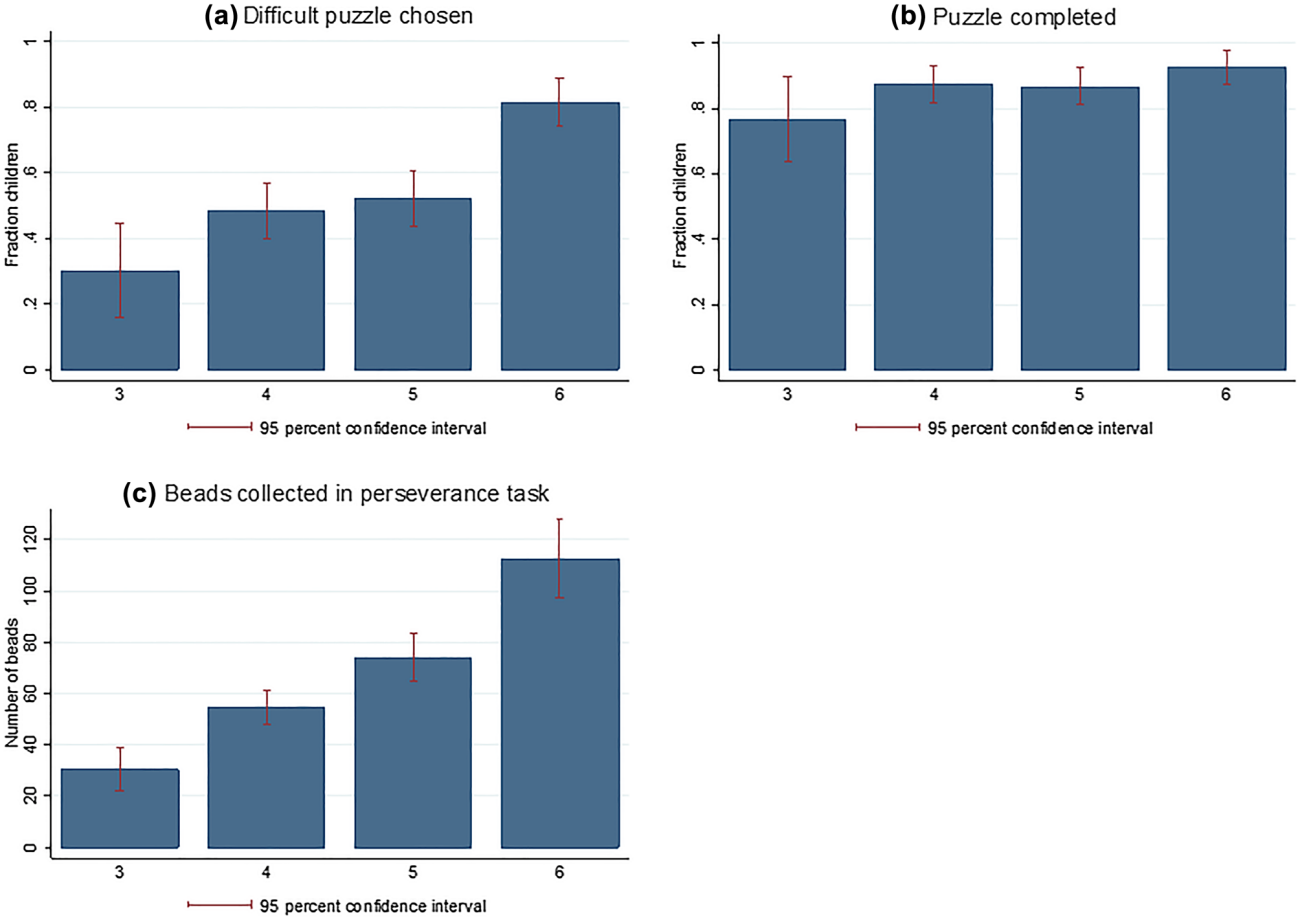
Behavior in the puzzle and the perseverance task by age. Notes: The upper left panel (**a**) shows the fraction of children who chose the more difficult puzzle. The upper right panel (**b**) presents the fraction of children who finished the puzzle that they had chosen by the end of our visit. The lower panel (**c**) indicates the performance of children in the perseverance task. All panels condition choices on age.

Figure 1 shows the relation of age to each of the three components of grit. Panel A presents the likelihood of challenging oneself by choosing the more difficult puzzle. While overall this likelihood is 56%, panel (a) demonstrates a strong increase with age. Recall that 3- and 4-year-olds, respectively 5- and 6-year-olds, had the same choices. In each pairwise comparison, we see a significant difference (*p* < 0.05 in each χ^2^-test). Looking at boys versus girls, we see that girls are significantly less likely to challenge themselves with the difficult puzzle (51% vs. 61%; *p* < 0.05; χ^2^-test).

Panel (b) of Fig. 1 indicates the fraction of children who followed through with the puzzle task and completed it before the end of our visit (to earn some additional tokens). Overall, 87% of children completed the puzzle, and again we see an increase in this likelihood with age (ranging from 77% for 3-year-olds to 93% for 6-year olds; *p* = 0.025; Cuzick’s Wilcoxon-type test for trend). The completion rate does not differ significantly between easy and difficult puzzles (86% vs. 89%).

Panel (c) of Fig. 1 shows the average number of beads that were collected by children in the perseverance task. The numbers increase monotonically from 3-year-olds to 6-year-olds (*p* < 0.001; Cuzick’s Wilcoxon-type test for trend). Girls sort significantly more beads than boys (80 vs. 66 on average; *p* < 0.05; Mann–Whitney-U-test). Figure S.3 in SI displays the average time spent on the task, contingent on age. It ranges from 5 min for the youngest cohort to 10 min for the oldest cohort (*p* < 0.001; Cuzick’s Wilcoxon-type test for trend), and the overall average is 7.9 min. By dividing the number of beads collected by the time spent on the task in each age cohort, one can see that productivity also increases by age (from around 6 beads per minute to about 11 beads; *p* < 0.001; Cuzick’s Wilcoxon-type test for trend).

Looking at behavior across the three choices, we can see that children who sort more beads are (i) more likely to choose the difficult puzzle (*p* < 0.001, Mann–Whitney-U-test) and (ii) more likely to follow through on their choice by completing the puzzle, irrespective of its difficulty (*p* < 0.001, Mann–Whitney-U-test). As we have already noted before, there is no significant correlation between the choice of puzzle and the likelihood of completing it. It is noteworthy also that none of these choices is significantly correlated with our measure of a child’s patience, i.e., the number of tokens saved for the next day in the time preference task. This is an indication that grit and patience (as measured here) are not related to each other, which matches earlier evidence for adults^7,20^. Interestingly, the decision to postpone the task to the next day, when children are given the choice to work on the perseverance task either today or tomorrow, can also be interpreted as a measure of patience. This alternative measure is available for the 241 children who could choose when to do this task. We find no significant correlation between postponing the task to the next day and the number of tokens saved for tomorrow in our time preference task (*p* > 0.16), which suggests that discounting may be domain specific rather than general.

**Figure 2:**
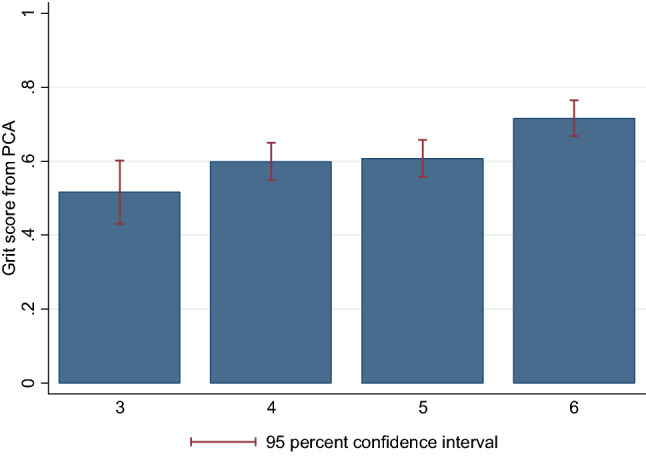
Grit score by age. Notes: The figure shows for each age cohort (on horizontal axis) the grit score from the principal component analysis (taking into account whether the difficult puzzle had been chosen, whether that puzzle had been completed, and whether a child performed above median in the perseverance task in its age group).

Next, we combine our three measures into a composite index of grit by using a principal components analysis (PCA). For this purpose, we use the two binary choices from the puzzle task and add a transformed measure from the perseverance task. We have seen that productivity in this task increases significantly with age, for which reason taking the number of beads collected would let older children appear grittier than they might be. We therefore split the sample within each age cohort at the median and use as our third component of grit whether a child belongs within its age cohort to the above- or below-median group with respect to the number of collected beads. From the PCA we construct a grit index and show it in Fig. 2 for each age cohort. We see a significant increase with age (*p* = 0.001; Cuzick’s Wilcoxon-type test for trend). This is confirmed in Table S.2 in SI where we see a significantly positive coefficient for age. Gender and the outcome measure for patience are insignificant. In that table we also control for the treatment variations in the perseverance task and note that children who choose voluntarily to work on the task today (when given the choice in the endogenous treatment) have a significantly higher grit score than the other children. This suggests that being willing to do a task on the spot—when one could delay it—is a strong signal of being grittier. In Table S.3 in SI we take into account family background characteristics. In total, 48% of parents returned the parental questionnaire, of which some did not answer all questions (in particular on income). So Table S.3 is based on a smaller data set. It confirms the positive influence of age on the grit score. In addition to that, it reveals a marginally significant coefficient of single parents on a child’s grit score and a positive correlation between parents’ assessment of their child’s grit and the actual grit score. The latter suggests that, in the aggregate, parents have a good intuition for their child’s level of grit.

When looking separately at our three components of grit—by running seemingly unrelated regressions on the number of beads collected, the likelihood to choose the difficult puzzle and to complete it (see Table S.4 in SI)—we find that age is significant for the total number of beads collected and for the likelihood to challenge oneself with the difficult puzzle, but not for the likelihood to complete the puzzle. We also control in these regressions for ability in the perseverance task and find that it matters for the total number of beads collected and for the likelihood to complete the puzzle. The level of enjoyment of the task, also elicited for the perseverance task, is weakly positively related to the performance in the perseverance task. Parents having a tertiary education is significantly positively related to the number of beads collected in the perseverance task, but reduces the likelihood to complete the puzzle task. Children from households with single parents (which account for 10% of all households) are more likely to challenge themselves with the more difficult puzzle. Household income raises the likelihood to choose the difficult puzzle as a larger challenge.

## Discussion

Grit is an important non-cognitive skill for educational and labor market success. Earlier work has concentrated on adults’ level of grit and how it relates to professional and personal life outcomes^6–9^, or on how to teach grit to primary school children^8^. Evidence on grit and its development in younger, preschool children has been lacking, however. We have shown with a sample of 3- to 6-year-old kindergarten children that the level of grit increases significantly in early childhood. For measuring grit, we have relied on a composite measure of perseverance, the willingness to challenge oneself and the ability to follow through on the challenge by meeting it successfully. Based on a parental questionnaire, we have found that parents have a good understanding of their children’s level of grit. Family background characteristics matter relatively little, even though parents’ education matters for perseverance and single parent status is indicative of the willingness to challenge oneself and of the composite grit score. One potential limitation of our grit score is that we do not consider any forms of setbacks or intermediate failures. For ethical reasons, we refrained from any such interference while children were working on their tasks, yet previous work has shown that coping with intermediate failures is important for success ^8^. How 3- to 6-year-old children react to such failures is certainly worth investigating in the future. We have not found a relationship between grit and our measure of time preferences. Given that experimentally elicited time preferences seem to develop in the direction of more patience in kindergarten and primary school^27^, we would have actually expected a positive correlation with grit as well. The lack of such a finding calls for more robustness checks in the future (despite previous work finding a similar pattern like ours for adults;^7,20^). Finally, further research would be welcome to understand why and under which conditions grit increases in the first years of one’s life. For example, parenting styles at home, or peer effects in kindergarten might play a role for the positive development of grit.

## Supplementary Information

Supplementary Information.

## Data Availability

Data will be made available in case of publication in a publicly accessible repository (Edmond of the Max Planck Society).
