# Supplementary material for: Grit increases strongly in early childhood and is related to parental background
Source: Sci Rep. 2022 Mar 3;12:3561. doi: 10.1038/s41598-022-07542-4 (PMC8894391; doi:10.1038/s41598-022-07542-4)
Supplement: Supplementary file 1 — Supplementary Information. [file 41598_2022_7542_MOESM1_ESM.pdf]

SUPPLEMENTARY INFORMATION (SI)

Grit increases strongly in early childhood and is related to parental background

Matthias Sutter, Anna Untertrifaller, and Claudia Zoller

Table S.1: Number of observations

age	male	female	total	% children answering all control questions correctly
3	21	22	43	91%
4	64	72	136	87%
5	73	69	142	91%
6	52	56	108	96%
	210	219	429	429

Table S.2: Grit score

Dependent variable: Grit score (PCA)	Coefficient (significance level)
Age (in years)	0.149***
Female (=1)	0.043
Impatience	0.021
Treatment exogenous tomorrow	–0.031
Treatment endogenous today	0.243**
Treatment endogenous tomorrow	–0.075
Constant	–0.493**
¤ #Observations	429

Notes: Age is measured in years. Impatience is measured as the sum of tokens consumed today (rather than saving them for tomorrow). The treatment with exogenously imposing the perseverance task to be performed on the first day of our visit ("today") is the benchmark for the other treatments. "Exogenous tomorrow" refers to children who were instructed about the task on the first day, but had to perform it on the second day. In the "endogenous" treatment, children could choose whether they wanted to do the perseverance task on the same ("endogenous today") or the next day ("endogenous tomorrow").

/* denotes significance at the 5%/1%-level.

Table S.3: Grit score and family background

Dependent variable: Grit score (PCA)	Coefficient (significance level)
Age (in years)	0.159**
Female (=1)	–0.050
Impatience	0.069
# of siblings	0.064
Single parent household	0.588*
Stay-at-home parent	0.184
Parent working part time	0.116
Parent with university degree	0.030
Parents' assessment of child's grit	0.040*
Household income (in brackets)	0.080
Constant	–1.215***

Notes: Age is measured in years. Impatience is measured as the sum of tokens consumed today (rather than saving them for tomorrow). Stay-at-home parent indicates at least one parent identified as staying at home with the children. Parent working part time means at least one parent worked part-time (rather than full-time). Household income was measured in brackets as monthly income after taxes (with categories €1,500 or less; €1,500-2,500; €2,500-3,500; more than €3,500; see SI).
*/**/*** denotes significance at the 10%/5%/1%-level.

Table S.4: Seemingly unrelated regressions on number of beads in perseverance task, and choice of puzzle and its completion

	Beads in perseverance task	Difficult puzzle chosen	Puzzle completed
Age (in years)	19.75***	0.55***	0.08
Female (=1)	10.87	–0.45*	0.24
Ability in perseverance task	4.70***	0.02	0.09*
Level of enjoyment of perseverance task (1-5)	¤ 7.73*	0.15	–0.09
Impatience	6.00	0.08	–0.06
# of siblings	–3.86	0.27*	–0.61**
Single parent household	–18.74	1.95***	–0.14
Parent staying at home	–6.41	0.82*	0.98*
Parent working part time	–4.71	0.17	0.13
Parent with university degree	22.22**	–0.26	–0.68**
Parents' assessment of child's grit	1.86	0.00	0.05
Household income (in brackets)	–6.91	0.40**	0.15
Constant	–59.09**	–4.42	0.06

Notes: Age is measured in years. Impatience is measured as the sum of tokens consumed today (rather than saving them for tomorrow). Stay-at-home parent indicates at least one parent identified as staying at home with the children. Parent working part time means at least one parent worked part-time (rather than full-time). Household income was measured in brackets as monthly income after taxes (see SI and notes to Table S.3).*/**/*** denotes significance at the 10%/5%/1%-level.

Figure S.1: Puzzle task to challenge oneself

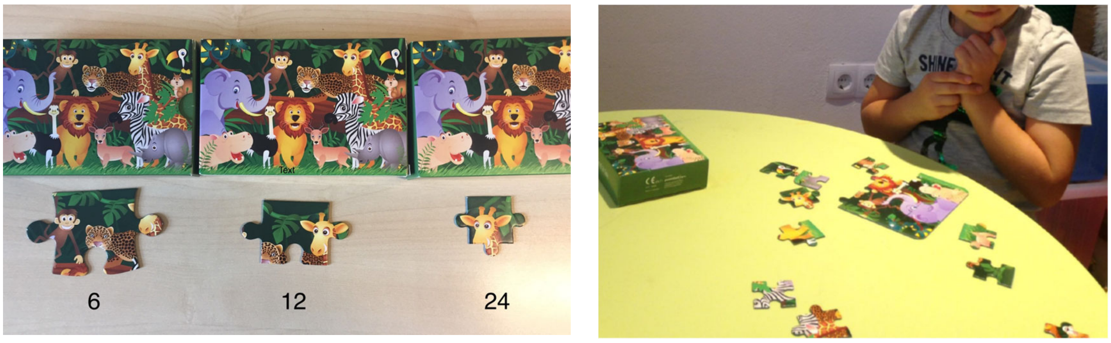

Notes: Children could choose between two different puzzles with the same picture which differed in the piece size and consequently the number of pieces. We presented children aged 3 and 4 with an easy puzzle with 6 pieces while the difficult puzzle entailed 12 pieces. 5 and 6 year-olds were given the option of an easy puzzle with 12 pieces and a difficult puzzle with 24 pieces. The experimenter showed the child the two identical puzzle boxes and took out one piece each to show the difference in piece size.

Figure S.2: Setup perseverance task

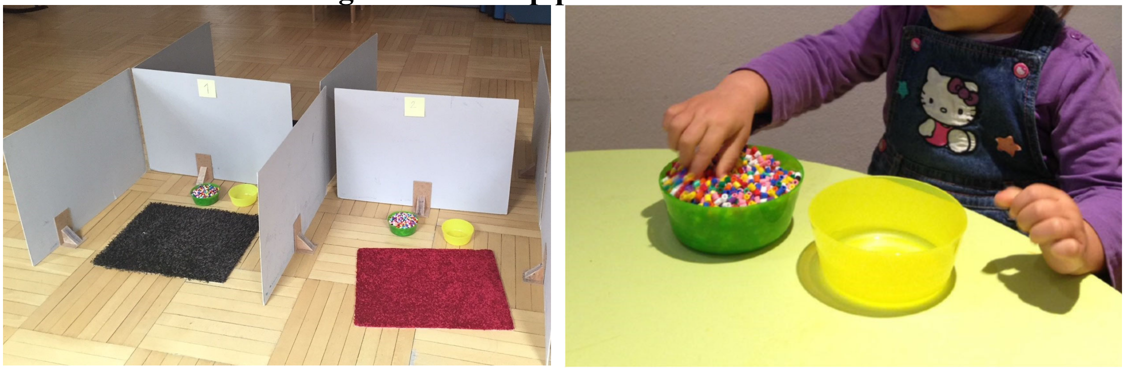

Notes: Children were seated in separate "cubicles" while working on the perseverance task. The task consisted of collecting the yellow beads out of the green bowl putting them into the yellow bowl.

Figure S.3: Time (in seconds) spent in the perseverance task by age cohort

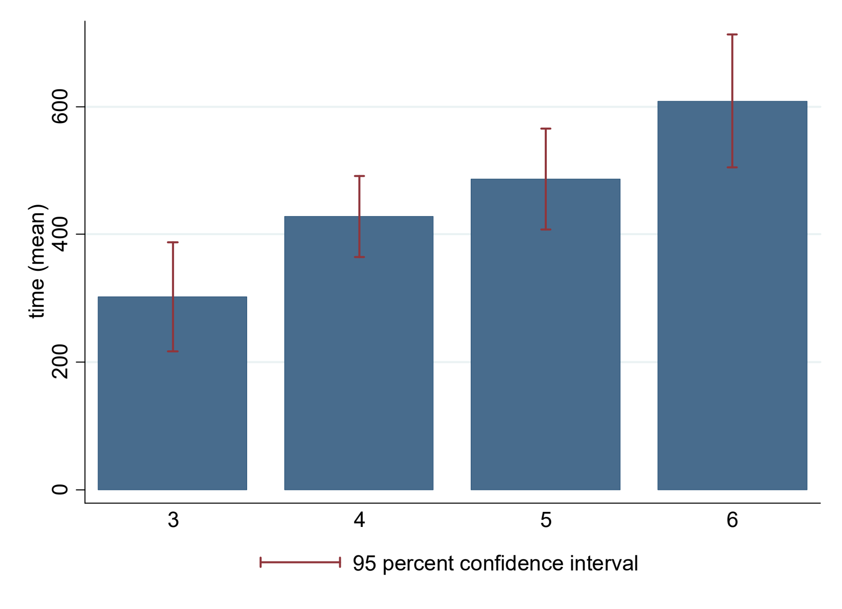

Notes: The figure shows the average time (in seconds; vertical axis) spent in the perseverance task, conditional on age (shown on the horizontal axis)

Instructions

Instructions translated from German. German instructions available upon request.

Hello! My name is .... *[name of experimenter]*. What's your name? Would you like to play a game with us?

[Experimenter and child walk to experimenter room, showing the presents at the entrance and sit down in dedicated space. Two bags with the child's name are prepared.] I will explain how the game works, so listen closely. In this game you can collect tokens which you can exchange for some presents afterwards. After I explain the game you will repeat it back to me, alright? And since you've been paying close attention I will already give you one token, which you can exchange for one present at the end of the game. Let's put the token over here and start the game. *[token placed in today bag]*

TIME PREFERENCES

In this game you can collect tokens for today and for tomorrow, which you can exchange for presents either today or tomorrow. Look, here I have pink and blue bowls. The tokens in the pink bowl can be exchanged into presents TODAY, the tokens in the blue bowl can be exchanged into presents TOMORROW.
Do you know what tomorrow means? Tomorrow means that you will sleep for one night to receive the tokens from the blue bowl and then you can exchange them into presents. I'm sure your kindergarten teacher has told you that we will be back tomorrow. We will be back tomorrow morning and also bring the same presents with us.

Now, the tokens from the pink bowl can be exchanged for presents today and the tokens from the blue bowl can be exchanged for presents tomorrow. Got it? Here we have three possibilities and you can pick one: *[show three sets of cardboards with bowls]*

- Option 1: If you choose option 1, there are 2 tokens in the pink bowl and none in the blue bowl. That means you will receive 2 presents today and no presents tomorrow.

- Option 2: If you choose option 2, there is 1 token in the pink bowl and 2 tokens in the blue bowl. That means you will receive 1 present today and 2 presents tomorrow.

- Option 3: If you choose option 3, there are no tokens in the pink bowl and 4 tokens in the blue bowl. That means you will receive no presents today and 4 presents tomorrow.

You may now choose one of these three options. But first please answer some questions:

- How many options can you choose? *[child: one]* • What happens if you choose option 1? How many tokens will you receive today and how many tokens will you receive tomorrow? *[let child count tokens in each bowl]*

- What happens if you choose option 2? How many tokens will you receive today and how many tokens will you receive tomorrow? *[let child count tokens in each bowl]*

- What happens if you choose option 3? How many tokens will you receive today and how many tokens will you receive tomorrow? *[let child count tokens in each bowl]*

[repeat instructions if child cannot answer correctly repeat up to two additional times] Control questions asked in randomized order for each experimenter.

Well done! Now please choose one option.
Great, you have chosen option ... . That means you will receive X tokens presents today and X presents tomorrow. Can you tell me why you chose this option? [note down answer] Let me put the tokens you will receive tomorrow into this bag. See, I wrote your name on it so I can save it for tomorrow. Tomorrow you will come back and exchange these tokens for presents. *[put tokens into today and tomorrow bags]*

PUZZLE TASK

[prepare puzzles: 3 and 4 year-olds - 6 and 12 pieces; 5 and 6 year-olds 12 and 24 pieces] You've done a great job so far! Would you like to play another game? Look, here I have two puzzles. Both puzzles have exactly the same picture. But one puzzle is more difficult and one puzzle is easier to do. This puzzle is difficult *[show puzzle with more pieces]*, because the puzzle pieces are smaller. This puzzle is easier because the puzzle pieces are bigger *[show one larger and one smaller puzzle piece for comparison]*. Do you see the difference? If you manage to do the difficult puzzle, you will receive two additional tokens. If you manage to do the easier puzzle, you will receive one additional token. You will have until lunch time to do the puzzle.

Now, before you decide which puzzle to keep, I have some questions for you.

- Which puzzle is more difficult?

- How many presents will you receive if you complete the easy puzzle? How many presents will you receive if you complete the difficult puzzle? • Until when do you have to do the puzzle to still receive your presents for this task?

[repeat instructions if child cannot answer correctly repeat up to two additional times] Great, now you can decide which puzzle you would like. You will be able to keep that puzzle and take it home with you afterwards.

PERSEVERANCE TASK

Well done! Now I have one last task for you. You can collect some additional tokens in this task, which you can again exchange for presents afterwards. Should I explain how the task works?
Look, here I have another bowl. There are many colorful beads in this bowl. The task is to collect ONLY YELLOW beads. The more yellow beads you collect, the more presents you will get. You can collect the yellow beads for as long as you want to. If you want to stop collecting beads, just say stop and we will count how many beads you have collected.

Depending on the number of yellow beads you collect you will receive a few or many presents. If you, for example, collect this many yellow beads - these are 20 beads - *[show bowl with 20 yellow beads]* you will receive one present.

Do you understand how the task works?

- What do you have to do? *[child: collect yellow beads]*

- If you collect many yellow beads, will you receive more presents or fewer presents? *[child: more]*

- When will you stop the task? *[child: when I want to]*

Great! So let's do a trial round to see how it works. You can start picking yellow beads from the bowl. Ready? Go! *[stop child after 30 seconds, note number of beads]*
Great job!

[Read assigned treatment only!]
T1a (today): Since you've done such a good job, you can sit down over there right now and start collecting yellow beads from the bowl. You can collect yellow beads for as long as you want to. If you want to stop collecting yellow beads signal to .... over there and she/he will count your beads and exchange your tokens into presents. **T1b (tomorrow):** Since you've done such a great job, you can do this task tomorrow. Tomorrow we will come back to this room and then you can sit in one of those spots over there and collect yellow beads. You will be able to collect yellow beads for as long as you want to. If you want to stop collecting yellow beads you will signal to ... over there and she/he will count your beads and exchange your tokens into presents tomorrow. *[repeat explanation of RET when child comes back the next day]*
T2 (endogenous): Since you've done such a great job, you can decide when you want to do this task. You can either do the task right now or you can do it tomorrow remember we will be back tomorrow with the same presents. For this task you will sit in one those spots over there and collect as many yellow beads as you want to. You can collect yellow beads for as long as you want to. If you want to stop collecting yellow beads signal to ... over there and she/he will count your beads and exchange your tokens into presents.
When would you like to do this task? Now or tomorrow? *[note down decision and read the according paragraph below]*

- You have decided to the task now. That means you can sit down over there and start collecting yellow beads. If you want to stop collecting yellow beads just signal to ... over there. Then you can exchange your tokens for presents afterwards.

- You have decided to the task tomorrow. That means we will pick you up tomorrow and take you to this room again. We're almost done for today! Now you get to exchange your tokens for presents and then I'll take you back to your class. Thank you for doing such a great job today!

[different experimenter is responsible for supervising perseverance task; child signals to stop the task, note down time and weigh beads on scale, convert into tokens]

You've done a great job! Can you tell me how much fun it was to collect beads? Look, here I have five smiley faces. This face is sad because it did not like the task at all. The face next to it didn't think it was that much fun either but not as bad as the first one. The face in the middle thought it was kind of ok. This face is smiling because it liked the task. And this face here is laughing a lot because it really liked the task. How much did you like the task? Can you show me the face that fits you the most?
Alright that's it for today! Now let's exchange your tokens for presents! Then I will take you back to your class (if applicable: and I'll see you again tomorrow). Thank you for doing such a great job today! *[exchange tokens into presents with child; put chosen presents into bags, add parent questionnaire and seal them; take child back to class and leave bag at child's spot in wardrobe]*

Parental questionnaire

Parental questionnaire translated from German. German version available upon request.

Dear parents, we kindly ask you to fill in this anonymous questionnaire. All answers are voluntary. Thank you for your collaboration!

Demographic information about your person

Gender:

☐ female
☐ male

Age:……….. years

Occupation:

☐ full-time job
☐ part-time job
☐ currently not employed

Highest educational degree:

☐ Mandatory schooling
☐ Vocational training
☐ High-School
☐ University (Bachelor/Master Degree)
☐ University (PhD)

Language, primarily spoken at your home (please indicate only one):

☐ German
☐ Turkish

☐ Serbian/Croatian
☐ other: ....

Number and age of children:

☐ 1 child, age: .... years
☐ 2 children, age: .... years & .... years
☐ 3 children, age: .... years & .... years & .... years
☐ 4 children or more, age: .... years & .... years & .... years & .... years & .... years

I’m raising my children:

☐ alone
☐ with my partner

Age of my partner: .... years

Partners occupation:

☐ full-time job
☐ part-time job
☐ currently not employed

Partners highest educational degree:

☐ Mandatory schooling
☐ Vocational training
☐ High-School
☐ University (Bachelor/Master Degree)
☐ University (PhD)

Net monthly income of our family (voluntary disclosure):

☐ below 1,500 Euro
☐ 1,500 Euro - 2,500 Euro
☐ 2,500 Euro - 3,500 Euro
☐ above 3,500 Euro

We live in a:

☐ rental flat/house
☐ own flat/house

Assessment of your child

In the following we will report several statements. Please indicate for each statement how well it describes your child.

1= not at all to 5= to a very high extent

If I ask my child to perform an unpleasant task he/she tries to postpone the task for as long as possible.	1	2	3	4	5
My child likes to dawdle.	1	2	3	4	5
My child likes to spend a lot of time on a given task.	1	2	3	4	5
My child gets easily distracted.	1	2	3	4	5
Whatever my child begins, he/she wants to finish.	1	2	3	4	5
It's hard for my child if he/she has to wait.	1	2	3	4	5
My child likes playing challenging games.	1	2	3	4	5

During our project in kindergarten, we asked children to choose between three alternatives:

1) Option 1: two presents today, nothing tomorrow.
2) Option 2: one present today, and two presents tomorrow. 3)
Option 3: nothing today, and four presents tomorrow.

We promised children to come back the next day with presents, which were as nice as the ones they could get on the first day. Independently of what your child might have chosen, which option would you prefer for your child? The decision you make now has no impact on your child.

Which option would you choose for your child?

☐ Option 1: two presents today, and no present tomorrow

☐ Option 2: one present today, and two presents tomorrow

☐ Option 3: no present today, and four presents tomorrow

We also played the following game with the children. Now we are interested in your opinion.

The children were told to collect only yellow beads from a bowl of small, multicolored beads. The children could autonomously decide for how long they wanted to work on this task and how many beads they collected. Moreover, children could decide if doing the task right away or postponing it to the next day.

Which option would you choose for your child?

☐ "What is done, is done." My child should do the task right away.

☐ "Better late than never." My child should take his/her time and conduct the task on the following day.

Thank you for your participation.
